# The expression of water channel proteins during human salivary gland development: a topographic study of aquaporins 1, 3 and 5

**DOI:** 10.1007/s10735-017-9731-6

**Published:** 2017-08-01

**Authors:** Fernanda de Paula, Tathyane Harumi Nakajima Teshima, Ricardo Hsieh, Milena Monteiro Souza, Claudia Malheiros Coutinho-Camillo, Marcello Menta Simonsen Nico, Silvia Vanessa Lourenco

**Affiliations:** 1 Department of Stomatology, School of Dentistry, University of Sao Paulo, Av Prof Lineu Prestes, 2227 Cidade Universitária, São Paulo, SP CEP 05508-000 Brazil; 2 Department of Dermatology, School of Medicine, University of Sao Paulo, São Paulo, Brazil; 3 Department of Pathology, AC Camargo Cancer Centre, São Paulo, Brazil

**Keywords:** Aquaporin, Human, Salivary gland, Development, Morphogenesis

## Abstract

Some members of aquaporin family (AQP) plays crucial functions in salivary synthesis and secretion. These proteins expression has already been reported during salivary gland formation, however no previous studies in human developing glands have been performed. We evaluated AQP1, 3 and 5 expression through the stages of human salivary gland morphogenesis and discuss the possible role of AQP for glandular maturation. Human salivary glands derived from foetuses aged between 14 and 25 weeks were submitted to immunohistochemistry. At the bud stage, membrane expression of AQP1, 3 and 5 were observed within the epithelial bud cells presenting a similar apicolateral pattern, also found at the pseudoglandular stage, present within the terminal portions of future acini, while AQP5 was also particularly strong at the apical membrane of pre-acinar and pre-ductal cells. AQP5 was co-localised with Cytokeratin 7. Similar AQP1, 3 and 5 expression were observed at the following canalicular stage, where distinct and strongly luminal and acinar AQP5 expression is present. During the final terminal bud stage, AQP1 was only identified in serous acini, myoepithelial and endothelial cells, while differentiated mucous acinar cells and ducts were negative. AQP3 was detected at apicolateral membranes of both mucous and serous acini. AQP5 also showed a diffuse expression in mucous and serous acini, in addition to strong apical membrane expression within lumen of intercalated ductal cells. This topographic analysis of AQP1, 3 and 5 revealed differences in the expression pattern throughout salivary gland developmental stages, suggesting different roles for each protein in human glandular maturation.

## Introduction

Human salivary glands are basically composed of acinar, ductal and myoepithelial cells, with the main role of producing and secreting saliva into the oral cavity to maintain the oral homeostasis. They are classified into major and minor glands, which produce different types of secretion according to the glandular components and the neuronal stimulation. While the minor salivary glands are exclusively composed of mucous cells, the major parotid glands secrete a watery secretion by serous cells. The major submandibular and sublingual glands in turn produce mixed saliva, as they are composed of mucous and serous acinar cells (Katchburian and Arana 2014; Delporte et al. 2016). The terminal secretory units of the salivary glands composed of acinar and myoepithelial cells secrete the salivary fluid into the lumen of intercalated ducts, moving towards striated and excretory ducts, where intense ionic changes between the extracellular matrix and the ductal cells to form the final saliva.

**Fig. 1:**
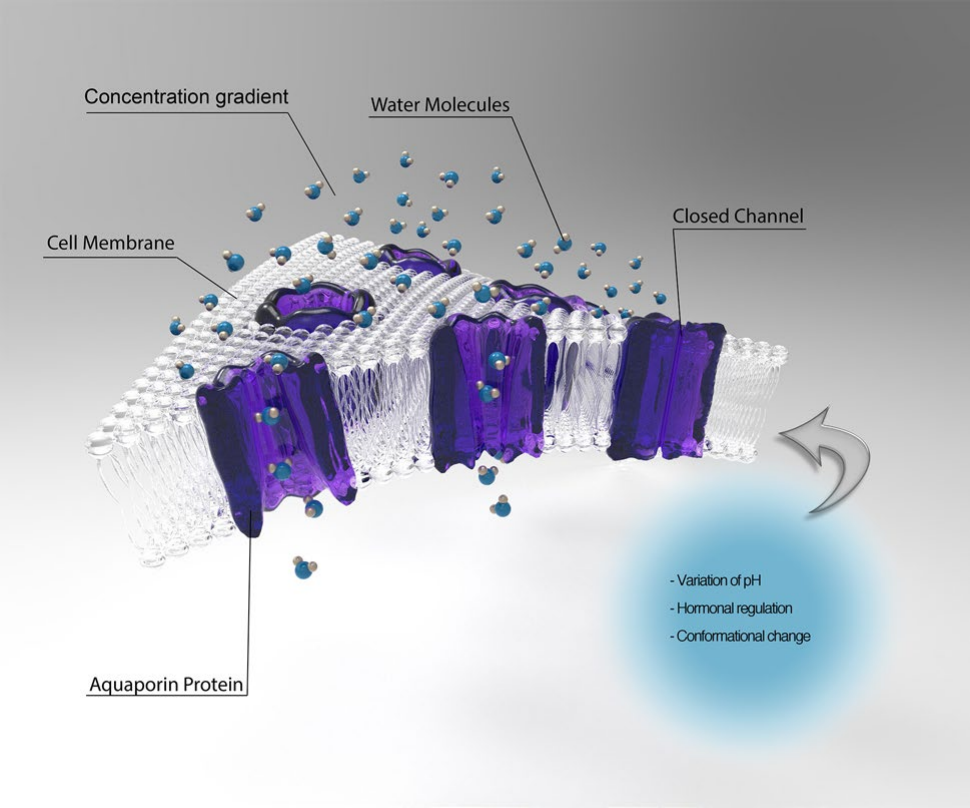
Schematic figure showing the cell localisation of water channel protein aquaporins. They are located through the plasma membrane in order to function as a conduct between outside and inside the cell. The transmembranic permeability of AQPs can occur through pH balance, hormonal regulation or conformational changes, allowing water and ion transport according to their concentration gradient

As saliva is a watery fluid, the amount of water reabsorption by acinar cells is important to define the salivary secretion, so as the basic mechanisms underlying this process. A transepithelial ionic gradient regulates this mechanism of fluid exchange by osmosis and diffusion through the basolateral and apical acinar cell membranes, which is facilitated by transmembranic water channel proteins known as aquaporins (Delporte et al. 2016; Kozono et al. 2002). This also depends on different isoforms of the aquaporins between the cell membrane and chemical expression levels of sodium potassium chlorine co-transporter, potassium chlorine co-transport, non-selective cation channel and volume regulated anion channel (Day et al. 2014). Aquaporins (AQP) are essential to provide the passage of water, electrolytes and small solutes across the phospholipid bilayer of the cellular plasma membrane (Fig. 1) in order to synthesise a balanced salivary composition (Kozono et al. 2002; Wang et al. 2015). The permeability of the pores of the cell membrane can be regulated in different pathways by aquaporins, however the specific signalling for each aquaporin member remains unclear. It has been suggested that some aquaporins ensure the regulation of membrane permeability through the influence of pH, hormonal regulation and changes in AQP pore conformation (Gonen and Walz 2006). Other studies in turn suggest that a tight junction-mediated paracellular mechanism is also able to contribute to salivary secretion between acinar cells alongside with the transcellular fluid exchange accomplished by aquaporins (Abe et al. 2016; Ding et al. 2017).

Upon the discovery of the first AQP, this protein was initially identified as CHIP28 and later as aquaporin 1, which was described by Peter Agre and colleagues in the late 1980s (Agre et al. 1987; Deen et al. 1994; Agre 2009). The aquaporin protein family is composed of 13 members (AQP 0 to 12) and classified into three subgroups: the classical aquaporins (AQP 0, 1, 2, 4, 5, 6 and 8), which are capable of carrying water; the aquaglyceroporins (AQP 3, 7, 9 and 10) that can carry water and small solutes like glycerol and urea; and the superaquaporins (AQP 11 and 12), which are recently reported to have unusual and uncertain transport roles (Ishibashi et al. 2014; Zhu et al. 2015; Moeller et al. 2016). Few isoforms of aquaporins were identified in adult glands within different subcellular compartments, potentially playing distinct roles in glandular function and maintenance (Gresz et al. 2001). Elucidating the molecular basis of aquaporin control mechanisms can lead to the understanding of the water movement across biological membranes in health and disease processes (King and Agre 1996).

The expression of AQP1, 3 and 5 has also been described during mouse salivary gland development, suggesting that aquaporins may also have an important role for the gland formation (Akamatsu et al. 2003; Larsen et al. 2009, 2011; Aure et al. 2011). This mechanism is driven by branching morphogenesis, which comprises complex and dynamic tissue interactions involving neuronal and endothelial cell interaction, cell proliferation, polarisation, differentiation epithelial-mesenchymal communication and cell death, resulting in a complex network of acinar bulbs and ducts (Cutler 1990; Patel et al. 2006; Lourenço and Kapas 2005; Lourenço et al. 2007; Teshima et al. 2016). No studies on human development have been reported yet, and this work aims to evaluate the expression pattern of AQP 1, 3 and 5 during human salivary gland morphogenesis, describing them according to each developmental stage and discussing the possible role for those proteins in this process compared to animal models.

## Materials and methods

### Tissue preparation

Major and minor salivary glands of 20 post-mortem human foetuses from natural miscarriages aged between 14 and 25 weeks were obtained from the School of Medicine of the University of São Paulo under the approval of the local Ethical Committee (protocol number 456.090). The glands were dissected from fixed tissue (formalin 10%), histologically processed, paraffin-embedded, serial-sectioned and stained with haematoxylin and eosin to firstly study their morphology. Selected specimens from major glands (parotid and submandibular) and minor glands were then submitted to immunohistochemistry in order to investigate the expression of AQP1, 3 and 5. The expression of each protein was analysed qualitatively, according to each salivary gland developmental stage, classically classified into initial bud, pseudoglandular, canalicular and terminal bud stages according to Tucker (2007).

### Immunohistochemistry

Serial section (4 µm) of developing salivary glands were deparaffinised, re-hydrated and submitted to antigen retrieval (citric acid, pH 6.0). The expression of each protein was investigated on all 47 samples, and the experiments were performed in triplicates to verify the reproducibility of the results. The sections were incubated in 3% aqueous hydrogen peroxide for 15 min to quench endogenous peroxidase activity, followed by Protein Block Serum-Free incubation (DakoCytomation, Carpinteria, CA, USA) for 20 min at room temperature to suppress nonspecific binding. Primary antibodies were incubated overnight at 4 °C (Abcam, AQP1 1:1500; AQP3 1:1000; AQP5 1:900; K7 1:50) then followed by incubation with the indirect dextran polymer detection system (En Vision—Dako, Carpinteria, CA, USA) or with secondary fluorescent antibodies for the double immunofluorescence (Alexa Fluor 488 and 568, Invitrogen, 1:300, and and TO PRO3, 1:1000, Invitrogen). Samples were coloured with 3′3 diaminobenzidine tetrachloride (DAB) for 3 min at room temperature and counterstained with Carazzi’s haematoxylin. Negative controls were incubated only with non-immune serum instead of the primary antibody, and positive controls were considered according to manufacturer’s datasheet recommendation.

Two experts in SG development performed a blind qualitative evaluation of each protein expression using a conventional optical microscope (Olympus E330), while the fluorescence images were captured utilising confocal laser microscopy (Zeiss LSM 510 META).

## Results

The expression of AQP1, 3 and 5 during human developing salivary glands was analysed according to each morphogenetic stage. The glandular phenotype was utilised for this analysis rather than the foetal age as developing human SG present a very heterogeneous formation with different stages found within the same gland. The results were therefore based on the conventional classification of salivary gland development previously described as initial bud, pseudoglandular, canalicular and terminal bud (Tucker 2007; Teshima et al. 2016; de Paula et al. 2017).

**Fig. 2:**
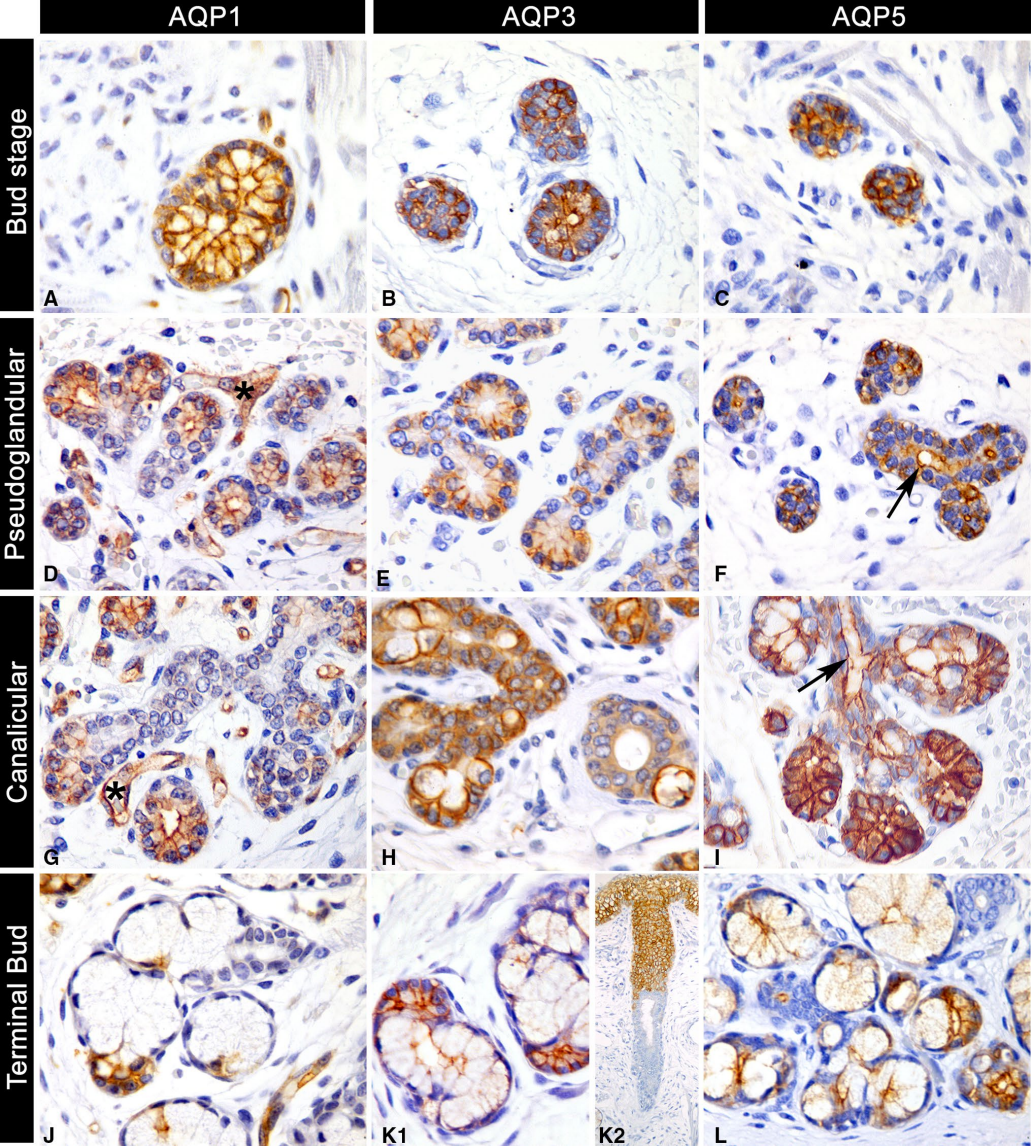
Proteins AQP1, 3 and 5 were present at all developmental stages of human foetal salivary glands. **a, b, c** *Initial bud stage* high expression of AQP1, 3 and 5 at the basolateral and apical membranes of solid epithelial buds; **d, e, f** *pseudoglandular stage* immunoexpression of AQP1 and 5 at the apicolateral membrane of glandular end buds (future acini) (**d, f**), while AQP3 was mostly basolateral in acini (**e**). Incipient ducts showed only presence of AQP5 at the luminal layer (**f**, *arrow*); **g, h, i** *canalicular stage* presence of AQP1 at the apicolateral membrane of secretory end buds. Ducts are negative for AQP1 and AQP3 (**g**, **h**), whereas AQP5 (**i**) is detected at the apicolateral membrane of epithelial cells of the glandular end buds and in the luminal layer of intercalated and interlobular ducts (**i**, *arrow*); **j, k, m** *terminal bud stage* AQP1 is observed at the apicolateral membrane of serous acinar cells. Endothelial cells of small capillaries are positive for AQP1 (**j**). AQP 3 (**k1**) is expressed at the apicolateral membrane of serous cells and at the apical pole of mucous acinar cells. Extralobular excretory ducts are negative for AQP 3. AQP 5 (**l**) is expressed at the apicolateral membrane of acinar cells and at the luminal surface of intercalated ducts. **k2** illustrates internal positive control on oral epithelial cells in contrast to negative excretory ducts for AQP3. Endothelial cells of small capillaries are consistently positive for AQP1 (**d, g**, *asterisks*). Magnification: **k2** 200×; **d**–**i, l** 400×; **a**–**c, j, k1** 630×

Overall, AQP1, 3 and 5 were detected during all stages of human salivary gland morphogenesis where they showed similar expression pattern in major and minor developing glands (Fig. 2), summarised in Table 1. They all presented membranous positivity in epithelial cells throughout the process, however AQP1 positive cells were also observed in myoepithelial cells and small capillaries in the glandular stroma (Fig. 2d, g asterisk). AQP1 and 5 were identified in the apicolateral membrane of epithelial cells forming the end buds, while the AQP3 was mainly expressed in the basolateral surface of these future acinar structures. Moreover, AQP5 was highly expressed within the luminal layer of ductal and acinar structures from pseudoglandular and canalicular stage (Fig. 2f, i, arrows).

**Table 1:** Summary of protein expression of AQP1, 3 and 5 according to each developmental stage of human foetal salivary glands

	AQP1		AQP3		AQP5	
	Acini	Ducts	Acini	Ducts	Acini	Ducts
Bud stage	Membrane	Absent	Membrane	Absent	Membrane	Absent
Pseudoglandular	Apicolateral membrane	Absent	Basolateral membrane	Absent	Apicolateral membrane	Luminal layer
Canalicular	Apicolateral membrane	Absent	Basolateral membrane	Absent	Apicolateral membrane	Luminal layer
Terminal bud	Serous glands	Absent	Serous/mucous	Absent	Serous/mucous	Proximal ducts

At the initial bud stage the expression of AQP1, 3 and 5 were similarly observed at the membrane of epithelial bud cells (Fig. 2a–c). During the pseudoglandular stage, the protein expression of AQP1 and 5 was detected at the apicolateral membrane of epithelial end buds (Fig. 2d, f), while only AQP3 was present at the basolateral membrane of these structures (Fig. 2e). Similar expression pattern was sustained during the following canalicular stage of glandular morphogenesis, where AQP1, 3 and 5 were found at the membrane of end bud regions (Fig. 2g, h, i). Regarding the ductal area, there was no positivity for AQP1 and 3 within the forming ducts (Fig. 2g, h), however AQP5 was strongly expressed at the apical cell membrane of newly formed and well-established ducts lining the lumen space (Fig. 2f, i, arrow).

At the final terminal bud stage, AQP1 was still not identified within any ductal structure, although was highly expressed within serous acinar cells (Fig. 2j). AQP3 was in turn detected at the basolateral membrane of developed serous and mucous acinar cells (Fig. 2k1). Collecting excretory ducts were, however, mostly negative as illustrated in Fig. 2k2, while the keratinocytes of the covering oral mucosa expressed high levels of AQP3 as expected (considered as internal positive control). AQP5 maintained its expression at the apicolateral membrane of acinar cells at this stage, where it was also present at the luminal layer of proximal developing ducts (Fig. 2l).

**Fig. 3:**
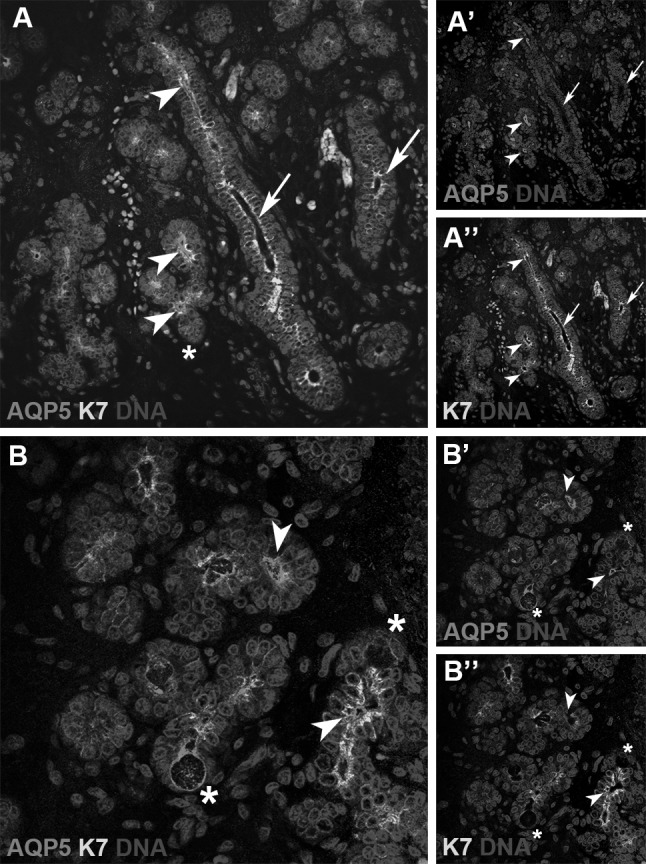
AQP5 was highly expressed within immature acini and presumptive ductal areas of developing submandibular glands. **a, a’, a’’** immunolocalisation of AQP5 and K7 during canalicular stage of human submandibular gland (200×). *Arrows* luminal expression of K7 where there is a clear luminal space already present, and AQP5 is completely absent; *Arrowheads* co-localisation of AQP5 and K7 in future ducts. **b, b’, b’’** Higher magnification of acinar region of human developing submandibular gland showing co-localisation (*arrowheads*) of AQP5 an K7 within forming ducts and exclusive AQP5 expression on differentiated acinar cells (*asterisks*) (400×)

AQP5 was the only aquaporin consistently found within the lumen space of developing ducts throughout gland morphogenesis. In order to address whether those AQP5 positive cells are future acini or in fact ducts, the expression of this protein in correlation with presumptive lumen marker cytokeratin 7 (K7) was analysed (Fig. 3). Mature ducts showing an expanded lumen space during gland development were K7 positive in the luminal layer and completely negative for AQP5 (Fig. 3a, a’, a’’, arrows). A striking co-localisation of AQP5 and K7 was however observed within presumptive duct structures, where both markers were concentrated mainly at the apical area (Fig. 3, arrowheads). Developing acini in turn were AQP5 positive only with an apicolateral expression in the epithelial cells (Fig. 3b, asterisks).

## Discussion

This study presented for the first time the immunoexpression of AQP1, 3 and 5 during the developmental stages of human salivary gland morphogenesis. Overall these proteins were strikingly positive at the acinar structures, and AQP5 was also observed within developing ducts throughout gland formation, which was then confirmed by double staining with a presumptive ductal marker. Aquaporins are essential for water transport and salivary secretion, and the presence of these proteins during gland development strongly suggests distinct roles during morphogenetic processes as previously shown in animal models. Although human salivary glands are histologically similar to SG of small mammals, they seem to bare subtle peculiarities. They develop more slowly and heterogeneously than SG of rodents, requiring therefore additional studies to analyse important differences between both models as suggested before (de Paula et al. 2017).

Reactivation of endogenous AQP1 has been investigated as a strong target to establish gland regeneration in injured glands by collateral irradiation of head and neck cancer patients (Wang et al. 2017). In fact, we observed an important expression of AQP1 during gland development that may contribute for the prenatal process. We have identified AQP1 mainly at the apicolateral membrane of epithelial cells throughout human salivary gland morphogenesis. This particular finding was however in contrast to other studies in adult human glands, which have shown AQP1 in presumably myoepithelial cells around acini and intercalated ducts (Beroukas et al. 2002; Gresz et al. 2001). Throughout development, we have also observed AQP1 in presumptive myoepithelial cells and within endothelial cells of small capillaries surrounding the salivary gland lobules. Preliminary studies in our group are currently on progress to address this question using double labelling for AQP1 and myoepithelial and endothelial markers (data not shown). They seem however not co-localised with the myoepithelium, which indicates that AQP1 is likely not specific to myoepithelial cells in human salivary gland morphogenesis and requires further investigation.

Studies in rat salivary glands described the immunolocalisation of AQP1 only in the microvasculature, which was also observed at prenatal stages, but no evidence of this protein in ductal or acinar structures (Li et al. 1994; Akamatsu et al. 2003). The expression pattern of AQP1 seem to be important for water transport from blood vessels to SG, aiding the formation of the future salivary fluid, corroborating our results (Akamatsu et al. 2003). In addition, recent studies on the role of vascularisation in mouse salivary glands have reported the requirement of endothelial cells for gland development by promoting expansion of specific progenitor cells during early stages (Kwon et al. 2017).

The expression pattern of AQP3 during human SG morphogenesis was also restricted to acinar structures throughout development as AQP1, however it was mostly found in the basolateral membrane of epithelial cells and it was completely negative in ducts and endothelial cells. In rat submandibular glands, Akamatsu et al. (2003) analysed the gene and protein expression of AQP3 during prenatal stages. In this study they reported a unique gene expression of AQP3, suggesting it plays a specific role in gland development, which is in agreement with our findings in human tissue. In rat adult glands, Nielsen et al. (1997) and King et al. (1997) found however no protein expression of AQP3 and weak presence of AQP1 in salivary glands.

Larsen et al. (2009) have also previously investigated the expression of AQP during salivary gland development however utilising the mouse model. They have demonstrated distinct RNA and protein expression pattern of AQP1, 3 and 5 amongst other family members, indicating that they might be associated with cell volume regulation, transepithelial transport, proliferation, cell death and other mechanisms, during development of submandibular glands. Furthermore, corroborating our results in humans, they suggested an important role of AQP3 during developing stages, while it decreased at early post-natal stages. In humans, the protein expression of AQP3 was found at the lateral membrane of mucous and serous acini of adult salivary glands, which was in agreement with our findings in foetal glands (Gresz et al. 2001).

Extensive studies have already suggested a fundamental role of AQP5 for SG development and homeostasis. AQP5 is reported to be the major protein involved in regulating the permeability of acinar cells and it therefore regulates the salivary ionic composition and the flow rate (Krane et al. 2001). In agreement to our results, the immunoexpression of AQP5 was reported at the apical membrane of rat submandibular pro-acinar cells (at late embryonic day 18) and during the formation of mature acini that became clearly distributed during the differentiation and establishment of mature acini (Akamatsu et al. 2003). Sugimoto et al. (2013) have also identified the expression of AQP5 in the apical membrane of serous acini of rat submandibular glands, in addition to the basolateral membrane of mucous acini.

Nielsen et al. (1997) have reported AQP5 expression at the apical membrane of acini and within intercalated ducts of adult rat submandibular glands. In accompanying studies utilising the same antibodies, AQP5 was found weakly at late developmental stage only from embryonic day 20, increasing postnatally (King et al. 1997). Embryonic glands were reported to show increasing expression of AQP5 through development in both studies, which was further supported by others (Akamatsu et al. 2003). In contrast, our results showed AQP5 positive cells at the apical membrane of acini and along the lumen space of intercalated and terminal ducts.

In addition to the acinar expression, Akamatsu et al. (2003) have also identified AQP5 positive cells in the SG vasculature as AQP1 in endothelial cells, and absent in intercalated ducts of developing rat submandibular glands. Moreover they have also reported to find less expressive AQP5 compared to AQP3 in rat salivary glands from late embryonic day 20 as also reported in human adult glands by Gresz et al. (2001). This finding may be valid for mature glands, however our results showed the opposite scenario, where the stronger and more broad distribution of AQP5 was much more suggestive to contribute to gland development than AQP3.

Aure et al. (2011) have shown the expression of AQP5 at the initial stages of mouse sublingual gland development, which was more organized towards birth and in young adult glands. In comparison, our results showed earlier detection of AQP5 in human developing glands, from the initial bud stage, while Aure et al. (2011) first observed a scattered pattern this protein at the canalicular stage of gland morphogenesis. Despite of expressing AQP5 earlier in development, human SG also showed a more consistent acinar and ductal positivity in more developed glands towards the terminal bud stage.

In larger mammals, Scocco et al. (2011) identified AQP5 in adult sheep mandibular and parotid salivary glands. In the parotid, they observed AQP5 at the lateral and apical membranes of acinar cells, also correlating its higher expression when feeding the animals according to water content food. There was also a difference in the expression of AQP5 between the types of acini, where serous cells showed higher positivity compared to mucous cells in submandibular glands, which were considered negative. Moreover, in contrast to human glands, AQP5 was not detected within any ductal structure in sheep glands.

Teymoortash et al. (2012) have compared the expression of AQP5 in human adult salivary glands with sialadenosis, revealing alteration of AQP5 in gland disease. In the normal control group, they revealed the presence of AQP5 strictly confined to the apical membranes of acinar cells, not reporting anything regarding the ducts. The abnormal localisation of AQP5 was correlated to the control of acinar cell volume, which likely contributes to salivary secretion. Further studies on human normal minor labial SG have shown similar pattern to Teymoortash et al. (2012), in which AQP5 was exclusively expressed in the apical region (Gresz et al. 2015). In this study, they have shown no expression of AQP5 in glandular labial ducts, however we also observed important positivity of this protein within rudimentary ducts of labial developing glands, similar to the major glands.

The presence of aquaporins in acinar structures during development is relatively supported by the main role of the water channel proteins to contribute to salivary production. However, the intriguing expression of AQP5 within rudimentary ducts in human foetal SG as shown with the double staining with K7 could possibly represent a distinct role for this protein during duct formation and more investigations are required. Recent studies have suggested the requirement of apoptosis for the very initial opening of the lumen space within the glandular duct (Teshima et al. 2016). Cell polarization and parasympathetic innervation are also contributing for lumen formation after pseudoglandular stage, showing defective ducts when depleted (Nedvetesky et al. 2014). It has been proposed that the presence of a controlled electrolytic flow within the lumen space contributes to its expansion during morphogenesis (Bagnat et al. 2010; Datta et al. 2011). Creating a cell turgor by hydrostatic pressure through activation of ductal channels has also been correlated to the lumen expansion (Sigurbjörnsdóttir et al. 2014), suggesting an additional role for AQP during development as raised before (Delporte and Steinfeld 2006).

This topographic description of the expression of AQP in human foetal salivary glands illustrated few important distinct results from those reported so far in different animal models. As many studies have shown no evidence of aquaporins during embryogenesis, our results raise questions regarding the importance of these aquaporins be expressed during early stages of development. Their particular expression pattern may indicate additional roles for these proteins during gland formation that have not been explored yet. Although the expression of AQP1 is widely reported within myoepithelial cells of adult SG in rodents, it remains difficult to categorically state that AQP1 is present within these cells in human developing glands. Similar analogy can be addressed to protein AQP5, which has shown a peculiar expression within developing intercalated ducts, also not previously reported. In addition, pilot experiments have been performed to investigate other types of aquaporins in human developing glands. The preliminary data have revealed the expression of AQP4 and 8, however requiring further investigation. Thus, our results bring new data in human glands that provide insights for further research to elucidate the role of these proteins during human salivary gland morphogenesis, as understanding the physiological characteristics of these proteins is essential for the selective regulation of the cell membrane. We also believe that identifying important differences between the expression of aquaporins in humans and animal models may raise new questions to be further investigations, also regarding new regenerative therapies, early biomarkers and potential diagnostic tools for glandular disease.
